# The relationship between home and community-based services utilization and self-reported quality of life for community-dwelling and assisted living residents with and without dementia

**DOI:** 10.1093/geroni/igaf118

**Published:** 2025-10-24

**Authors:** Eric Jutkowitz, John F Mulcahy, Peter Huckeldt, Mark Woodhouse, Stephanie Jarosek, Chanee D Fabius, Wyatt Tarter, Jack M Wolf, Tetyana P Shippee

**Affiliations:** Division of Health Policy & Management, University of Minnesota School of Public Health, Minneapolis, Minnesota, United States; Division of Health Policy & Management, University of Minnesota School of Public Health, Minneapolis, Minnesota, United States; Division of Health Policy & Management, University of Minnesota School of Public Health, Minneapolis, Minnesota, United States; Division of Health Policy & Management, University of Minnesota School of Public Health, Minneapolis, Minnesota, United States; Division of Health Policy & Management, University of Minnesota School of Public Health, Minneapolis, Minnesota, United States; Department of Health Policy and Management, Johns Hopkins Bloomberg School of Public Health, Baltimore, Maryland, United States; Biostatistical Design and Analysis Center, Clinical and Translational Science Institute, University of Minnesota, Minneapolis, Minnesota, United States; Division of Biostatistics & Health Data Science, University of Minnesota, Minneapolis, Minnesota, United States; Division of Health Policy & Management, University of Minnesota School of Public Health, Minneapolis, Minnesota, United States

**Keywords:** Aging in place, Caregiving, Long-term care services and supports

## Abstract

**Background and Objectives:**

Home and community-based services (HCBS) intend to allow individuals to age in their home or a home-like environment. The relationship between receiving specific types of HCBS and person-reported HCBS quality remains unclear.

**Research Design and Methods:**

We linked data on HCBS quality from 1413 respondents of the 2018 Minnesota National Core Indicators-Aging and Disability survey with claims from the Minnesota Department of Human Services Medicaid Management Information System. Among this sample of HCBS users, we used linear regression to evaluate the association between using specific types of HCBS (home health services; non-medical transportation; personal care assistant services; case management; adult day care; durable medical equipment/home modifications; and homemaker services and person-centered HCBS quality). Our regression models included an interaction between HCBS utilization and whether a respondent had a diagnosis of dementia. We stratified analyses between people living in the community and assisted living.

**Results:**

Using personal care assistant services was associated with significantly higher-quality scores among community-dwelling HCBS users. Using home health care, durable medical equipment/home modifications, non-medical transportation, homemaker services, and adult day care were not significantly associated with quality scores among HCBS users. On average, HCBS users with dementia reported lower quality scores than their counterparts without dementia. Among assisted living residents, quality was lower for people with dementia who did not use non-medical transportation or case management; however, this difference was not significant among users of these services. In the assisted living sample, analyses of quality subdomains found significant associations with service use that were not present when evaluating overall quality scores.

**Discussion and Implications:**

Personal care assistant services may be associated with higher quality among people in the community because this service supports activity of daily living and provides social interaction.

Innovation and Translational Significance The relationship between receiving specific types of home and community-based services (HCBS) and person-reported HCBS quality is unclear. We linked data on HCBS quality from the Minnesota National Core Indicators-Aging and Disability survey with claims from the Minnesota Department of Human Services. Using personal care assistant services was associated with significantly higher quality scores among a sample of community-dwelling HCBS users. In a sample of assisted living residents, receiving durable medical equipment/home modifications was associated with significantly higher overall quality.

## Introduction

By 2030, 20% of the US population will be over 65 years of age.^1^ Nearly 70% of people over age 65 will use long-term services and supports as they age, and 35% will use nursing home care for at least 1 year.^2^ The need for long-term care services and supports is driven by cognitive impairment, disability, functional limitations, and complex chronic conditions.

Most people want to age in their community or in home-like environments (eg, assisted living) rather than a nursing home.^3^^,^^4^ Home and community-based services (HCBS), which can include non-medical transportation and home health care, aim to promote the independence, health, and well-being of clients in the community.^5^ The ultimate goal of HCBS is to help people safely age in the community with a high quality of life. Over the past 30 years, public funders (eg, Medicaid) have invested in expanding HCBS, and state Medicaid programs now spend more of their long-term care budget on community-based services than nursing home care.^6^^,^^7^ More recently, Medicaid HCBS have expanded to provide services in assisted living and other congregate settings (excluding nursing homes), either through waivers or state plans. HCBS expansion efforts have also focused on building tailored supports for people living with complex conditions such as dementia, who represent nearly one-third of Medicaid HCBS users.^8^

Although HCBS is provided as an alternative to nursing home care, the effect of these services on healthcare utilization and quality is uncertain.^8^ Relative to nursing home care, HCBS shifts the responsibility of care to individuals and their family caregivers. Unlike nursing home care, which is provided 24/7, HCBS is scheduled (eg, personal care provided for set hours during the week), and services may not provide the intensity of care that people need or prefer. Some data suggest that compared to nursing home residents, HCBS users are more likely to use acute health services (eg, emergency departments).^9^ Further, among older adults receiving HCBS, those with dementia are more likely than those without dementia to experience hospitalizations.^8-10^ One scoping review of HCBS satisfaction and quality found that reliable and respectful care providers and person-centered models of care are associated with higher person-reported quality. Service interruptions and unmet needs were drivers of lower quality HCBS.^11^ Data on HCBS quality were primarily limited to small program evaluations, and overall, the review concluded that little is known about the ­relationship between the type of HCBS utilization and person-reported quality.

The National Quality Forum framework operationalizes high-quality HCBS as being “person-centered,” with the goal of improving consumer outcomes and promoting community living.^11-13^ Building on the National Quality Forum HCBS quality framework, the National Core Indicators-Aging and Disability (NCI-AD) is one survey that states can adopt to assess the performance of publicly funded long-term care. The Minnesota Department of Human Services was an early adopter of NCI-AD and started administering the survey in 2015. To make NCI-AD data actionable and to monitor the quality of state-funded HCBS, the Minnesota Department of Human Services developed and validated quality indices from NCI-AD survey responses.^14^^,^^15^

In this study, we link data from the 2018 Minnesota NCI-AD survey to administrative claims data to examine the association between using specific types of HCBS (eg, adult day care; obtained via claims data) and person-centered HCBS quality (obtained via NCI-AD). Although we are unable to determine a causal relationship between using specific types of HCBS and quality, we account for time bias (ie, simultaneous causality) by examining types of HCBS utilization (independent variable) up to 15 months before a person participates in the NCI-AD (used to obtain quality outcome). First, we explore the relationship between using specific types of HCBS and person-reported quality. Second, we explore differences in person-centered quality for HCBS users with and without dementia. We expect that quality will be lower for HCBS users with dementia based on data that caring for people with dementia is more demanding than providing care for people without cognitive impairment.^16-18^ For example, people living with dementia require substantially more long-term care services than older adults without dementia but with other similar health needs.^19-21^ Importantly, we stratify analyses between assisted living residents and people in other community housing settings. Although assisted living is increasingly housing Medicaid recipients, little is known about the HCBS used by this population.

## Methods

### Data sources

#### NCI-AD

We obtained HCBS quality measures from NCI-AD data. The NCI-AD survey is administered in partnership between ADvancing States, Human Services Research Institute, and state agencies to evaluate the quality and availability of HCBS to consumers living in the community, assisted living, other congregate living settings, or nursing homes. The surveys are cross-sectional and conducted in person. Respondents or a proxy (for respondents unable to participate in the survey) answer standardized measures on quality of life, health outcomes, and system performance (eg, service coordination, access, and choice). State participation in NCI-AD is voluntary. Since 2015, approximately 23 states have participated in NCI-AD.

We limited our analysis to the 2017-2018 Minnesota NCI-AD survey respondents who were ≥65 years of age and enrolled in the Medicaid Elderly Waiver program because (1) Minnesota is the only state where NCI-AD surveys can be linked with administrative claims (described below), (2) people in the Medicaid Elderly Waiver are community dwelling (the waiver excludes nursing home residents) and enrolled in Medicaid, which enables us to obtain HCBS utilization from claims data, (3) 2017-2018 survey waves were conducted before the COVID-19 pandemic, and (4) previous work has developed and validated quality indices using NCI-AD Minnesota data. The full 2017-2018 Minnesota NCI-AD survey sampled individuals 65 years and older from the Alternative Care program, State Plan-funded home care, and the Elderly Waiver program. The Alternative Care program enrolls people who need nursing home-level care but who live in the community and are not eligible for Medicaid. State-funded home care provides short-term care for people moving from the hospital or nursing home back to their home. The Elderly Waiver program provides HCBS to people living in the community who need nursing home-level care and are eligible for Medicaid. Additional details on the sampling frame and survey process can be found in the NCI-AD 2017-2018 Minnesota Results report.^14^

#### Medicaid management information system

We used HCBS utilization indicators derived from the Minnesota Department of Human Services Medicaid Management Information System (MMIS). Specifically, we queried the MMIS eligibility files (which define enrollment in Medicaid and in specific waivers), the recipient file (for demographic data), and claims data sets. We organized the individual claims into categories based on the Minnesota Department of Human Services category of service codes, which is used to group individual claims into higher-level categories of HCBS (eg, non-medical transportation). Per our data use agreement, these data were aggregated at the person-month level. The summary MMIS file contained person-month level HCBS utilization (based on the category of service codes) from January 2017 up to the month each participant’s NCI-AD survey was collected (February 2018-June 2018), which resulted in a median follow-up time of 15 months between HCBS utilization and the measurement of HCBS quality. The analytic file also included administrative data on basic demographics (age, race, marital status, and plan enrollment) and comorbidities.

### Participants

We linked the 2017-2018 Minnesota NCI-AD respondents to monthly MMIS data. The linked files included 3787 persons. We excluded 2338 NCI-AD respondents under age 65 or who were not enrolled in the Medicaid Elderly Waiver program. We also excluded 36 respondents who answered <5 of the items necessary to calculate quality (described below). Our final analytic sample included 1413 Minnesota Medicaid Elderly Waiver NCI-AD respondents with complete claims data.

### Measures

#### MMIS measures (independent variables)

The summary MMIS file contained monthly person level indicators (yes or no) for 7 HCBS categories: (1) home health services, (2) non-medical transportation, (3) personal care assistant services, (4) case management, (5) adult day care, (6) durable medical equipment (DME)/modifications (or just DME for the assisted living clients), and (7) homemaker services (combined chore services, companion services, meals, caregiver training, family support, and homemaker services). For each HCBS category, we divided the total number of months a participant used a service by their total months of follow-up. These proportion variables (ie, the proportion of months a service was used) were the primary independent variables.

#### HCBS quality measures (dependent variable)

We used the Minnesota Department of Human Services validated quality index derived from NCI-AD to create an outcome measure.^15^ The overall index is comprised of 3 domains derived from the 2018 NCI-AD survey: (1) security (scored 0 to 10), (2) self-determination (scored 0 to 14), and (3) care experience (scored 0 to 8). NCI-AD respondents answer each question, which is described in detail in Supplementary Table 1. Higher scores indicate better quality, and when necessary, we reverse-coded items. The overall HCBS person-reported quality index is the sum of the scores from the individual items and ranges from 0 to 32 for self-respondents and from 0 to 10 for people with a proxy respondent. Quality was evaluated as a proportion (individual score divided by maximum possible score) to enable the inclusion of both self-respondents and proxy respondents in our analyses. To account for missing item data, the maximum possible score was calculated for each subject based on the number of items they completed. The original index included a fourth domain for physical function, but we excluded this domain because the items inquired about a participant’s self-identified disability status and need for assistance with activities of daily living. These items reflect a participant’s health status as opposed to satisfaction with service quality for HCBS. Excluding these items (and domain) is consistent with established definitions of quality of life in long-term care, which suggest that health status and functional status are correlates of quality of life but should not be used to directly measure quality of life.^22^

#### Covariates

We obtained the following covariates from MMIS administrative data: respondent age (65-74, 75-84, and 85+), race/ethnicity (non-Hispanic White, non-Hispanic Black, Hispanic, other), and 16 chronic condition indicators (brain injury, cancer, congestive heart failure, chronic obstructive pulmonary disorder, stroke, depression, diabetes, hard of hearing, hypertension, liver disease, mental health disorder, myocardial infarction, obesity, peripheral vascular disorder, kidney disease, and serious mental illness). We also extracted from MMIS whether an individual had dementia, which is based on the presence of a medical diagnosis for dementia in their Medicaid administrative claims (hospital and outpatient medical records). We also created indicator variables for the health plan of each participant (ie, an indicator for enrollment in 1 of 8 health plans). Specifically, Minnesota has managed Medicaid, and beneficiaries select among health plans that are offered at the county level. We also determined the total number of HCBS (0-7) a person ever received during the period of observation. We obtained marital status (married, unmarried) and whether a respondent had a proxy respondent from the NCI-AD survey. Marital status is an important covariate because having a spouse can represent a built-in support system that may further complement HCBS. The presence of a proxy respondent is an important covariate because it indicates a respondent's level of morbidity.

### Statistical analysis

We descriptively evaluated the characteristics of the sample overall and stratified by place of residence (community and assisted living). We estimated the adjusted association (stratified by place of residence) between the proportion of each type of HCBS used and dementia status (independent variables) and HCBS quality (outcome variable). We included an interaction between HCBS utilization and whether a respondent had a diagnosis of dementia. We estimated separate regressions for each measure of HCBS utilization due to collinearity between measures. To account for concurrent service use and to isolate the association of each service with quality, we controlled for the total number (0-7) of HCBS a person ever received during the period of observation. All analyses also included covariates specified above (demographic characteristics and each comorbidity) and the participant’s health plan as fixed effects. HCBS use (proportion of months using a service) was treated as a continuous variable, and we report analyses by comparing people who do and do not use the service for all months. Using these regression models, we estimated the adjusted mean HCBS quality score stratified by dementia status (yes vs no) and HCBS use (use in all months vs no use). Our primary comparison of interest is between people with and without dementia who use a specific HCBS, and between people with and without dementia who do not use a specific HCBS. We also explored the association between HCBS utilization and each of the 3 domains of quality separately (ie, each domain as an outcome measure).

Analyses of each domain were performed on the subset of respondents who completed at least one item from the domain. Because very few assisted living residents reported using personal care assistants (*n *= 8 [1.4%]) or adult day care services (*n *= 17 [3%]), we did not estimate regression models with these independent variables for people in assisted living. All models were estimated using generalized estimating equations with a working exchangeable correlation structure at the county level to account for potential differences in local service availability. All hypothesis tests were 2-sided at the 5% significance level; no adjustments were made for multiple comparisons. Analyses were conducted using Stata version 17 and R version 4.4.2.

## Results

Of the 1413 people in the Minnesota Medicaid Elderly Waiver NCI-AD sample, 40.6% were assisted living residents and 59.4% were living in a community setting (Table 1; Supplementary Appendix Table 2 reports NCI-AD survey month). Overall, most respondents identified as White (75%) and were unmarried (86%). People with dementia (*n *= 482), compared to people without dementia (*n *= 931), were older (age 85 + 40% vs 28%), more likely to be men (29% vs 24%), and more likely to be White (78% vs 74%; Supplementary Table 3).

**Table 1.** Characteristics of Minnesota Medicaid Elderly Waiver 2017-2018 National Core Indicators-Aging and Disability (NCI-AD) respondents.

Characteristics	Full sample (*N *= 1413)	Community-dwelling (*n *= 839)	Assisted living (*n *= 574)
**Age group, *n* (%)**			
** 65-74**	411 (29%)	310 (37%)	101 (18%)
** 75-84**	548 (39%)	364 (43%)	184 (32%)
** 85+**	454 (32%)	165 (20%)	289 (50%)
**Female, *n* (%)**	1050 (74%)	622 (74%)	428 (75%)
**Race, *n* (%)**			
** African American/Black**	160 (11%)	152 (18%)	8 (1.4%)
** White**	1059 (75%)	532 (63%)	527 (92%)
** Hispanic**	135 (9.6%)	120 (14%)	15 (2.6%)
** Other**	59 (4.2%)	35 (4.2%)	24 (4.2%)
**Marital status, *n* (%)**			
** Married**	130 (9.2%)	102 (12%)	28 (4.9%)
** Unmarried**	1221 (86%)	727 (87%)	494 (86%)
** Unknown/refused**	62 (4.4%)	10 (1.2%)	52 (9.1%)
**HCBS service utilization, mean (*SD*)^a^**			
** Home health**	0.29 (0.40)	0.40 (0.44)	0.11 (0.23)
** DME/modifications**	0.65 (0.38)	0.70 (0.38)	0.57 (0.37)
** Non-medical transport**	0.29 (0.37)	0.34 (0.39)	0.21 (0.31)
** Personal care assistant**	0.17 (0.36)	0.28 (0.43)	0.00 (0.04)
** Case management**	0.39 (0.36)	0.34 (0.37)	0.47 (0.34)
** Homemaker services**	0.43 (0.45)	0.66 (0.42)	0.09 (0.24)
** Adult day**	0.10 (0.28)	0.16 (0.34)	0.02 (0.11)
**Proxy respondent, *n* (%)**	81 (5.7%)	30 (3.6%)	51 (8.9%)
**Comorbidities, *n* (%)**			
** Brain injury**	64 (4.5%)	39 (4.6%)	25 (4.4%)
** Cancer**	236 (17%)	153 (18%)	83 (14%)
** Congestive heart failure**	483 (34%)	270 (32%)	213 (37%)
** Chronic obstructive pulmonary disease**	700 (50%)	452 (54%)	248 (43%)
** Stroke**	443 (31%)	273 (33%)	170 (30%)
** Dementia**	482 (34%)	190 (23%)	292 (51%)
** Depression**	762 (54%)	454 (54%)	308 (54%)
** Diabetes**	678 (48%)	451 (54%)	227 (40%)
** Hard of hearing**	564 (40%)	357 (43%)	207 (36%)
** Hypertension**	1240 (88%)	755 (90%)	485 (84%)
** Liver disease**	180 (13%)	128 (15%)	52 (9.1%)
** Mental health**	990 (70%)	601 (72%)	389 (68%)
** Myocardial infarction**	263 (19%)	177 (21%)	86 (15%)
** Obesity**	534 (38%)	383 (46%)	151 (26%)
** Peripheral vascular disease**	613 (43%)	329 (39%)	284 (49%)
** Kidney disease**	484 (34%)	284 (34%)	200 (35%)
** Serious mental illness**	158 (11%)	110 (13%)	48 (8.4%)
**Quality, mean (*SD*)^b^**			
** Overall**	0.85 (0.14)	0.86 (0.13)	0.83 (0.16)
** Security domain**	0.89 (0.19)	0.90 (0.17)	0.86 (0.22)
** Self-determination domain**	0.85 (0.17)	0.86 (0.16)	0.82 (0.19)
** Care experience domain**	0.79 (0.27)	0.81 (0.25)	0.76 (0.29)

a Proportion of months of service use.

b Individual quality was evaluated as a person’s quality score divided by the total possible score. Overall quality = self-report (range 0-32) and proxy (0-10), security = self-report (range 0-10) and proxy (0-0), self-determination = self-report (range 0-) and proxy (0-4), care experience = self-report (range 0-8), and proxy (0-6).

Abbreviations: DME = durable medical equipment; HCBS = home and community-based services.

Among the full sample, DME/home modifications were the most frequently used service in at least 1 month (90%), followed by case management (63%) and non-medical transport (56%). Notably, while all Elderly Waiver participants are assigned a case manager, there is no requirement for monthly case management. Consequently, some recipients may only interact with their case manager a few times per year. Home health care, personal care assistant services, non-medical transportation, adult day care, DME/home modifications, and homemaker services were used by more people living in the community compared to those in assisted living.

In the full sample, the mean (*SD*) overall quality was 0.85 (0.14). Overall quality was higher for people without dementia than for people with dementia (0.86 [0.14] vs 0.83 [0.16]; Supplementary Appendix Table 2). On average, quality was higher for people with dementia who had a proxy compared to self-report (0.90 vs 0.82). In contrast, quality was lower for people without dementia who had a proxy compared to self-report (0.82 vs 0.86; Supplementary Table 4).

### Association between type of HCBS utilization and overall quality

#### Community-dwelling

Table 2 shows the regression coefficients for the association between using HCBS and overall quality. Among community-dwelling respondents who use HCBS, using personal care assistant services was associated with significantly higher overall person-reported quality scores (0.02, 95% confidence interval [CI]: 0.00, 0.05; Supplementary Table 5). Using home health, DME/home modifications, non-medical transport, case management, home care, and adult day care were not significantly associated with overall quality.

**Table 2.** Association between using HCBS and overall person-reported HCBS quality.

Service use (proportion of months)	Community-dwelling (*n *= 839)		Assisted living resident (*n *= 574)	
	B (95% CI)	*p*	B (95% CI)	*p*
**1. Home health services**	−0.01 (−0.04, 0.01)	.29	0.07 (−0.01, 0.15)	.07
**Dementia (no = 0, yes = 1)**	0.00 (−0.03, 0.03)	.82	−0.02 (−0.05, 0.02)	.35
**Dementia × Home health services**	0.00 (−0.05, 0.05)	.94	−0.05 (−0.15, 0.05)	.31
**2. Durable medical equipment/home modifications**	−0.01 (−0.04, 0.01)	.40	0.04 (0.00, 0.09)	.07
**Dementia (no = 0, yes =1)**	−0.01 (−0.03, 0.02)	.64	−0.01 (−0.06, 0.04)	.61
**Dementia × DME/modifications**	0.00 (−0.03, 0.04)	.80	−0.02 (−0.09, 0.05)	.61
**3. Non-medical transportation**	−0.01 (−0.04, 0.01)	.37	−0.08 (−0.15, −0.02)	.01**
**Dementia (no = 0, yes =1)**	−0.01 (−0.03, 0.01)	.36	−0.04 (−0.08, 0.00)	.04*
**Dementia × Transportation**	0.02 (−0.01, 0.05)	.19	0.09 (0.01, 0.17)	.03*
**4. Personal Care Assistant Services**	0.02 (0.00, 0.04)	.02*	NA	
**Dementia (no = 0, yes =1)**	−0.01 (−0.03, 0.02)	.47		
**Dementia × Personal care assistant services**	0.02 (−0.03, 0.06)	.40		
**5. Case management**	0.03 (−0.02, 0.07)	.25	−0.08 (−0.14, −0.02)	.01**
**Dementia (no = 0, yes =1)**	0.01 (−0.01, 0.03)	.54	−0.08 (−0.13, −0.03)	.002
**Dementia × Case management**	−0.03 (−0.08, 0.03)	.32	0.12 (0.04, 0.19)	.002**
**6. Homemaker services**	0.01 (−0.02, 0.04)	.65	−0.06 (−0.14, 0.01)	.09
**Dementia (no = 0, yes =1)**	−0.01 (−0.04, 0.01)	.35	−0.03 (−0.07, 0.01)	.13
**Dementia × Homemaker services**	0.02 (−0.02, 0.06)	.43	0.07 (−0.04, 0.18)	.20
**7. Adult day care**	0.02 (−0.02, 0.06)	.35	NA	
**Dementia (no = 0, yes =1)**	0.00 (−0.02, 0.02)	.73		
**Dementia × Adult day**	0.00 (−0.03, 0.04)	.85		

Seven separate regressions were estimated in which the outcome was overall quality (individual quality score divided by total possible score) and the primary independent variables were service use, dementia and the interaction between service use and dementia. All regressions controlled for sex, age group, race/ethnicity, marital status, health plan, and chronic conditions. Coefficients for HCBS use are modeled on the proportion scale and represent the contrast between no use (ref) versus use in all months. People in assisted living received durable medical equipment but not home modifications.

* *p *< .05.

** *p *< .01.

*** *p *< .001.

Abbreviations: DME = durable medical equipment; HCBS = home and community-based services; NA = not available.

**Figure 1.**
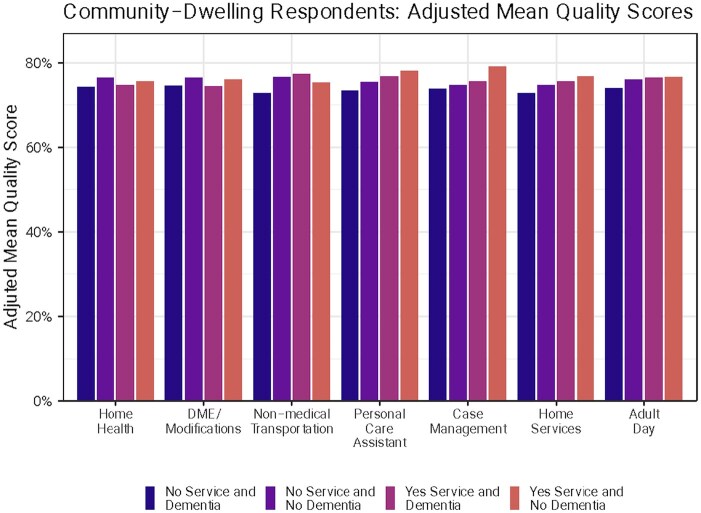
Community-dwelling: overall HCBS quality by dementia and HCBS use. Notes. HCBS = home and community-based services. The figure presents the adjusted mean quality measures from the regression and decomposes the interaction term into individual components. There was no significant difference in quality scores between people with and without dementia who used each service. There was no significant difference in quality scores by dementia status for people who did not use each service.

Figure 1 shows the overall adjusted mean quality by type of HCBS use (use in all months or no use) and dementia status (yes or no). The interaction term in Table 2 is the difference in differences between service use in all months and no service use between people with and without dementia. Among people who did not use HCBS, person-reported quality was lower (not statistically significant) for all services for people with dementia compared to people without dementia (coefficient range −0.01 to 0.00).

**Figure 2.**
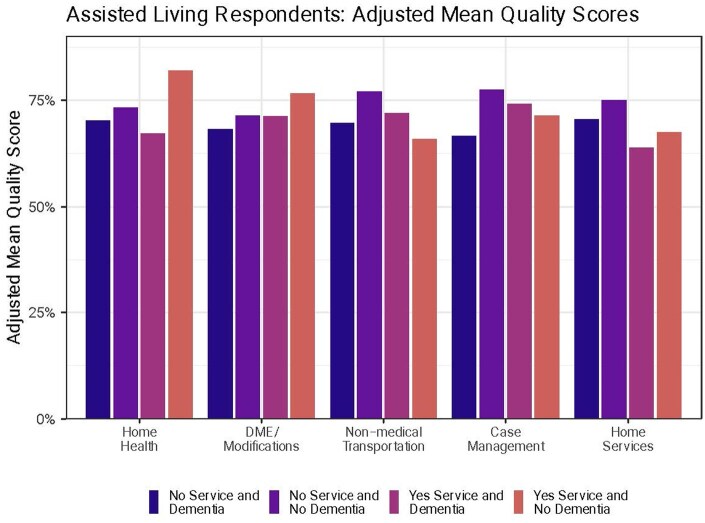
Assisted living: overall HCBS quality by dementia and HCBS use. Notes. HCBS = home and community-based services. The figure presents the adjusted mean quality measures from the regression and decomposes the interaction term into individual components. Quality was significantly lower for people with dementia compared to those without dementia who did not use non-medical transportation (−0.042, 95% CI: −0.081, −0.003) and case management (−0.077, 95% CI: −0.127, −0.028). There was no significant difference in quality scores between people with and without dementia who used each service.

Among people who used home health, DME/home modifications, and case management services, people with dementia had lower mean person-reported quality scores (not statistically significant) compared to those without dementia (coefficient range −0.02 to 0.00). Quality was higher, but not statistically significant, for people with dementia compared to those without dementia who used non-medical transportation, personal care assistants, and home care services (coefficient range 0.00-0.01). No interaction terms were statistically significant.

#### Assisted living

Among assisted living residents, using DME was associated with significantly higher overall quality (0.03, 95% CI: 0.00, 0.07; Supplementary Table 5). Receiving non-medical transportation, case management, and homemaker services was associated with lower (not statistically significant) overall quality. There was no other meaningful or statistically significant association between the other types of HCBS utilization and HCBS quality.

Among assisted living residents who did not use specific HCBS, overall quality was significantly lower for people with dementia compared to those without dementia who did not use non-medical transportation (−0.042, 95% CI: −0.081, −0.003; Figure 2) and case management (−0.077, 95% CI: −0.127, −0.028). Quality was also lower (not statistically significant) for people with dementia who did not use home health (−0.016, 95% CI: −0.005, 0.018), DME (−0.012, 95% CI: −0.060, 0.035), or home services (−0.03, 95% CI: −0.065, 0.010).

HCBS quality scores were lower (not statistically significant) for assisted living residents with dementia who used home health services (−0.067, 95% CI: −0.166, 0.032) or DME (−0.030, 95% CI: −0.078, 0.018) than their counterparts without dementia. Quality was higher (not statistically significant) for people with dementia compared to those without dementia who used non-medical transportation (0.046, 95% CI: −0.027, 0.118), case management (0.038, 95% CI: −0.009, 0.085), and home services (0.042, 95% CI: −0.059, 0.143).

### Association between type of HCBS utilization and domains of HCBS quality

#### Community-dwelling

Among community-dwelling respondents, there were no statistically significant or meaningful associations between HCBS utilization and the care experience domain (Table 3 and Supplementary Figure 1). Person-reported quality was significantly higher on the security domain for people who used personal care assistants (0.04, 95% CI: 0.02, 0.06). However, there was no significant difference in the security domain between service users by dementia status and non-users by dementia status (Supplementary Figure 2). Finally, person-reported quality scores were significantly lower on the self-determination domain for people who used DME/home modifications (−0.03, 95% CI: −0.06, −0.01) but higher for people who used personal care assistant services (0.04, 95% CI: 0.02, 0.07) and adult day services (0.04, 95% CI: 0.00, 0.08). Yet, there were no significant differences in the self-determination domain between service users and non-users by dementia status (Supplementary Figure 3).

**Table 3.** Association between using HCBS and domains of person-reported HCBS quality.^a^

Variable	Community-dwelling (*n *= 839)						Assisted living resident (*n *= 574)					
	Security^b^ (*n *= 830)		Self-determination^b^ (*n *= 838)		Care experience^b^ (*n *= 811)		Security^b^ (*n *= 561)		Self-determination^b^ (*n *= 574)		Care experience^b^ (*n *= 558)	
	B	*p*	B	*p*	B	*p*	B	*p*	B	*p*	B	*p*
**Home health services**	−0.02 (−0.04, 0.00)	.10	−0.03 (−0.06, 0.00)	.093	0.02 (−0.02, 0.06)	.37	0.00 (−0.11, 0.11)	>.99	0.04 (−0.03, 0.12)	.26	0.21 (0.10, 0.33)	<.001***
**Dementia (no = 0, yes = 1)**	−0.01 (−0.04, 0.03)	.73	−0.01 (−0.04, 0.03)	.74	0.00 (−0.05, 0.05)	.96	−0.03 (−0.06, 0.01)	.14	0.01 (−0.04, 0.05)	.73	−0.07 (−0.14, −0.01)	.021*
**Dementia × Home health services**	0.01 (−0.03, 0.06)	.57	0.01 (−0.04, 0.06)	.77	−0.02 (−0.11, 0.07)	.65	0.02 (−0.11, 0.16)	.73	−0.03 (−0.15, 0.09)	.63	−0.14 (−0.29, 0.01)	.061
**DME/Home modifications^c^**	0.01 (−0.03, 0.05)	.70	−0.04 (−0.07, −0.01)	.005 **	0.02 (−0.03, 0.07)	.40	−0.02 (−0.08,0.04)	.54	0.05 (0.00, 0.10)	.048*	0.12 (0.02, 0.23)	.022*
**Dementia (no = 0, yes =1)**	0.01 (−0.02, 0.04)	.46	−0.01 (−0.05, 0.02)	.45	−0.03 (−0.12, 0.05)	.44	−0.03 (−0.09, 0.03)	.33	0.00 (−0.06, 0.06)	.97	−0.06 (−0.16, 0.04)	.26
**Dementia × DME/Modifications**	−0.02 (−0.07, 0.03)	.48	0.02 (−0.04, 0.07)	.53	0.04 (−0.09, 0.16)	.57	0.01 (−0.08, 0.10)	.79	0.01 (−0.08, 0.09)	.90	−0.06 (−0.19, 0.06)	.34
**Non-medical transportation**	−0.01 (−0.04, 0.02)	.50	−0.01 (−0.04, 0.02)	.59	−0.02 (−0.07, 0.04)	.53	−0.09 (−0.17, −0.01)	.030*	−0.10 (−0.19, −0.02)	.021*	−0.06 (−0.14, 0.02)	.12
**Dementia (no = 0, yes =1)**	0.00 (−0.03, 0.03)	.97	−0.02 (−0.04, 0.01)	.28	−0.01 (−0.05, 0.04)	.72	−0.06 (−0.10, −0.01)	.009**	−0.01 (−0.06, 0.04)	.75	−0.10 (−0.16, −0.04)	<.001***
**Dementia × Transportation**	0.00 (−0.04, 0.05)	.96	0.04 (−0.03, 0.10)	.28	0.00 (−0.11, 0.11)	>.99	0.17 (0.08, 0.26)	<.001***	0.05 (−0.06, 0.16)	.41	0.05 (−0.11, 0.21)	.54
**Personal care assistant services**	0.04 (0.02, 0.07)	.002	0.04 (0.01, 0.06)	.002	−0.03 (−0.09, 0.04)	.42	NA		NA		NA	
**Dementia (no = 0, yes =1)**	0.01 (−0.03, 0.04)	.72	−0.02 (−0.05, 0.01)	.29	−0.02 (−0.06, 0.03)	.45						
**Dementia × Personal care assistant services**	−0.02 (−0.07, 0.02)	.31	0.04 (−0.01, 0.10)	.15	0.03 (−0.09, 0.14)	.66						
**Case management**	0.01 (−0.06, 0.08)	.84	0.02 (−0.04, 0.07)	.53	0.07 (−0.03, 0.16)	.16	−0.08 (−0.15, −0.01)	.017*	−0.11 (−0.19, −0.04)	.002**	−0.03 (−0.16, 0.10)	.69
**Dementia (no = 0, yes =1)**	0.01 (−0.04, 0.05)	.72	0.01 (−0.02, 0.03)	.68	0.00 (−0.07, 0.07)	.98	−0.07 (−0.11, −0.02)	.004**	−0.06 (−0.12, 0.00)	.039*	−0.14 (−0.23, −0.06)	<.001***
**Dementia × Case management**	−0.03 (−0.12, 0.07)	.59	−0.03 (−0.09, 0.04)	.41	−0.02 (−0.18, 0.14)	.77	0.09 (0.02, 0.16)	.017*	0.13 (0.05, 0.21)	.001**	0.11 (0.00, 0.22)	.055
**Homemaker services**	0.01 (−0.03, 0.06)	.54	0.01 (−0.02, 0.05)	.50	0.00 (−0.03, 0.03)	.87	−0.14 (−0.26, −0.02)	.027*	−0.01 (−0.10, 0.08)	.85	−0.10 (−0.19, 0.00)	.052
**Dementia (no = 0, yes =1)**	0.00 (−0.03, 0.03)	.89	−0.01 (−0.05, 0.02)	.43	−0.02 (−0.07, 0.03)	.48	−0.04 (−0.07, 0.00)	.026*	0.00 (−0.04, 0.05)	.89	−0.09 (−0.16, −0.02)	.008**
**Dementia × Homemaker services**	0.01 (−0.04, 0.05)	.80	0.02 (−0.03, 0.08)	.42	0.02 (−0.08, 0.11)	.75	0.17 (0.02, 0.32)	.029*	0.02 (−0.13, 0.17)	.80	−0.02 (−0.20, 0.15)	.81
**Adult day care**	0.04 (−0.02, 0.09)	.18	0.04 (0.00, 0.09)	.074	−0.05 (−0.12, 0.02)	.19						
**Dementia (no = 0, yes =1)**	0.00 (−0.03, 0.03)	.94	0.00 (−0.02, 0.02)	.91	−0.01 (−0.05, 0.03)	.57						
**Dementia × Adult day**	−0.01 (−0.06, 0.05)	.76	−0.01 (−0.08, 0.06)	.75	0.02 (−0.06, 0.11)	.61						

a Separate regressions were estimated in which the outcome was quality domain and the primary independent variables were service use, dementia, and the interaction between service use and dementia.

b Domain quality was evaluated as an individual quality score divided by the total possible score. Overall quality = self-report (0-32) and proxy (0-10), security = self-report (0-10) and proxy (0-0), self-determination = self-report (0-14) and proxy (0-4), care experience = self-report (0-8) and proxy (0-6).

c People in assisted living received durable medical equipment but not home modifications.

* *p *< .05.

** *p *< .01.

*** *p *< .001.

Abbreviations: DME = durable medical equipment; HCBS = home and community-based services; NA = not available.

#### Assisted living

Among assisted living residents, overall quality scores were significantly higher on the care experience domain for people who used home health (0.14, 95% CI: 0.02, 0.26) or DME (0.09, 95% 0.02, 0.17) but lower for people who used home services (−0.11, 95% CI: −0.21, −0.01). Quality scores on the care experience domain were significantly lower for people with dementia compared to their counterparts without dementia who did not use home health (−0.075, 95% CI: −0.138, −0.011), non-medical transportation (−0.102, 95% CI: −0.162, −0.043), case management (−0.143, 95% CI: −0.226, −0.059) and home services (−0.090, 95% CI: −0.157, −0.023; Supplementary Figure 4). Among service users, quality scores on the care experience domain were significantly lower for people with dementia compared to their counterparts without dementia who used home health (−0.217, 95% CI: −0.351, −0.082) or DME/home modifications (−0.119, 95% CI: −0.188, −0.049). There were no other significant associations on the care experience domain between people with and without dementia who used HCBS services.

Overall quality scores on the security domain were lower (not statistically significant) for people who used DME (−0.01, 95% CI: −0.06, 0.03) non-medical transportation (−0.01, 95% CI: −0.08, 0.06), case management (−0.04, 95% CI: −0.10, 0.03), and home services (−0.05, 95% CI: −0.13, 0.03). Quality scores on the security domain were significantly lower among assisted living residents with dementia who did not use non-medical transportation (−0.058, 95% CI: −0.101, −0.014), case management (−0.066, 95% CI: −0.110, −0.021) and home services (−0.039, 95% CI: −0.073, −0.005) compared to their counterparts without dementia (Supplementary Figure 5). Quality scores were significantly higher on the security domain for people with dementia who used non-medical transportation (0.111, 95% CI: 0.036, 0.185) compared to their counterparts without dementia. Significant interactions were detected bet­ween dementia and both non-medical transportation services, case management services, and home services.

Overall quality scores on the self-determination domain were significantly higher for people who used DME (0.05, 95% CI: 0.02, 0.09) and were significantly lower for people who used non-medical transportation (−0.08, 95% CI: −0.14, −0.01). Quality scores on the self-determination domain were significantly lower among assisted living residents with dementia who did not use case management (−0.059, 95% CI: −0.115, −0.003) compared to their counterparts without dementia (Supplementary Figure 6). Simultaneously, quality scores on the self-determination were significantly higher for people with dementia who used case management compared to their counterparts without dementia (0.074, 95% CI: 0.015, 0.132). These two differences were significantly different from one another. There were no other meaningful associations between service use and quality of self-determination.

## Discussion

In this study, we examined the association between using specific types of HCBS and person-reported HCBS quality. We found that using personal care assistant services was associated with higher person-reported quality among a sample of people living in the community who use any HCBS. There were nonsignificant differences in quality between people with and without dementia and those who did and did not use a service. Importantly, we provide some of the first data on HCBS and quality in assisted living, which is increasingly housing Medicaid recipients enrolled in HCBS waivers.^23^ Among assisted living residents, we found that quality was significantly higher for people who used DME/home modification. In addition, quality scores were significantly lower for assisted living residents with dementia compared to their counterparts without dementia who did not use non-medical transportation and case management, and these associations were different for residents living with dementia. Using personal care assistant services was associated with higher person-reported quality among people living in the community compared to HCBS users who did not use this service. Several reasons may explain these findings. First, personal care assistant services support activities of daily living and enable people to live in their homes longer. Second, a personal care assistant may provide engagement and social interaction. Social engagement is important and can increase quality of life.^24^^,^^25^ Third, differences in recall of the services used may be an important factor because evaluation of HCBS quality occurred after the use of HCBS. For example, people may be more likely to remember a personal care assistant, which requires more active engagement compared to a service that is used infrequently.

We stratified analyses by place of residence (community or assisted living) because assisted living residents have built-in services that may mimic important aspects of HCBS. Findings on the association between quality, dementia, and use of HCBS for assisted living residents are complicated. We found quality was lower for assisted living residents with dementia who did not use non-medical transportation and case management compared to their counterparts without dementia. However, among users of non-medical transportation or case management, there was no significant difference in quality between people with and without dementia. This suggests that using these services may be associated with a uniform level of quality irrespective of cognitive function, and people with dementia may have received a greater benefit (in terms of quality) from using HCBS because they had lower baseline quality scores.

Living with dementia was associated with significantly lower person-reported quality among assisted living residents, and the magnitude of this association was larger than in the community. As 51% of assisted living residents in our study were living with dementia, more work is needed to understand why people living with dementia experience lower quality despite making up most residents.

In the assisted living sample (but not in the community-dwelling sample), the analysis of quality domains identified significant associations between HCBS use/non-use and quality that differed from those observed for the overall quality score. Generally, the directionality of findings remained consistent, but the subdomains became statistically significant. For example, among service users, there were no significant differences in overall quality domains between people with and without dementia. However, on the care experience domain, quality was significantly lower for people with dementia compared to those without dementia who used home health and DME/home modifications. Conversely, in the security domain, quality was significantly higher for people with dementia compared to those without dementia who used non-medical transportation. One potential explanation for these findings may be that dementia affects only specific domains while others contribute to statistical noise.

## Limitations

Several limitations should be considered. First, we were unable to determine a causal relationship between the type of HCBS utilization and person-reported quality. We account for time bias by examining the type of HCBS utilization in the period before people reported quality; however, other factors (eg, health and social support) may confound the observed relationships. Second, our findings may not generalize to the broader US population of people receiving publicly funded HCBS, including people <65 years of age. We limited our analyses to the State of Minnesota, which ranked as #1 on the 2023 AARP Long-Term Services Support State Score Care,^20^ and used a quality index that was validated using Minnesota NCI-AD data. Related, we limited our analysis to individuals enrolled in the Elderly Waiver program, which is a population that must meet the need for at least a minimum level of care (ie, require nursing home level care) to qualify for the waiver. In addition, the design and delivery of specific HCBS varies widely between states and commonly even within a single state’s program. Third, we examined the association between overall quality and each type of HCBS service type separately rather than all HCBS services together. This was due to collinearity between the types of services, but we did control for total services used. Related, some of the services we evaluated may not directly align with all items in the quality measures, potentially leading to an underestimation of the association between service utilization and quality. Fourth, we evaluated quality as a proportion rather than on the original scale. This approach allowed us to include both people with and without proxy respondents; however, it may present challenges in interpreting the measure. Fifth, we used the Minnesota Department of Human Services category of service codes to group individual claims into categories of HCBS. Some of the HCBS categories are broad and can include a range of services. For example, homemaker services can include a housekeeper or companion services. An important next step is to examine the combination of services used by people and how the combination corresponds with person-reported quality. Finally, there is a need to evaluate the quality of HCBS for people <65 years of age.

## Conclusion

Among a sample of community-dwelling HCBS recipients, using personal care assistant services was associated with large and significantly higher overall quality scores. Using home health services, DME/home modifications, and non-medical transport, homemaker and adult day care were not associated with overall person-reported quality scores among a sample of community-dwelling people who use HCBS. There was no significant difference in overall quality score (or the subdomains) between community-dwelling people with and without dementia who used each type of HCBS. Finally, in a sample of assisted living residents, overall quality scores were lower for people with dementia who did not use non-medical transportation or case management. However, among those who used non-medical transportation, case management, or home services, there was no significant difference in quality of life between people with and without dementia. Importantly, in the assisted living sample, analyses of quality subdomains found significant associations with service use that were not present when evaluating overall quality scores.

## Supplementary Material

igaf118_Supplementary_Data

## Data Availability

We used data from the National Core Indicators-Aging and Disability survey and the Minnesota Department of Human Services. Investigators can contact the corresponding author for information about how to apply for access to the data used in our analyses. The study was not preregistered.
