# Supplementary material for: The relationship between home and community-based services utilization and self-reported quality of life for community-dwelling and assisted living residents with and without dementia
Source: Innov Aging. 2025 Oct 24;9(11):igaf118. doi: 10.1093/geroni/igaf118 (PMC12659963; doi:10.1093/geroni/igaf118)

***Innovation in Aging* Supplementary Material: Jutkowitz et al. The Relationship Between Home and Community-Based Services Utilization and Self-Reported Quality of Life for Community-Dwelling and Assisted Living Residents with and without Dementia**

**Supplementary Table 1.** HCBS Quality Measure Domains, Survey Questions, and Scoring

| **Domain and Survey Questions** | **Scoring** |
| --- | --- |
| *Security Domain: 5 Questions (scored 0 to 10)* | |
| 1. Are you ever worried for the security of your personal belongings? | 0 = no/never  2 = yes, at least sometimes |
| 2. In the last 12 months, has anyone used or taken your money without your permission? | 0 = no  2 = yes |
| 3. Do you feel safe at home/where you live? | 0 = no, rarely, or never  2 = yes, always, or most of the time |
| 4. Would you prefer to live somewhere else? | 0 = no  1 = maybe  2 = yes |
| 5. In general, do you like where you are living right now? | 0 = no  1 = in-between, most of the time  2 = yes |
| *Self-determination Domain: 7 Questions (scored 0 to 14)* | |
| 1. Can you eat your meals when you want to? | 0 = no, never  1 = somedays, sometimes  2 = yes, always, or almost always |
| 2. Do you get up and go to bed when you want to? | 0=no, never 1=somedays, sometimes  2=yes, always, or almost always |
| 3. Do people ask your permission before coming into your home/room? | 0 = sometimes/rarely, or never  1 = usually, but not always  2 = yes, always |
| 4. Do you like how you usually spend your time during the day? | 0 = no, never  1 = somedays, sometimes  2 = yes, always, or almost always |
| 5. Do you have access to healthy foods like fruit and vegetables if you want them? | 0 = no, never  1 = sometimes  2 = yes, often |
| 6. In general, do you get to do things you enjoy outside of home as much as you want to? | 0 = no  2 = yes |
| 7. Do you have transportation when you want to do things outside of your home/where you live, like visit a friend, go for entertainment, or do something for fun? | 0 = no  1 = sometimes  2 = yes |
| *Care Experience Domain: 4 Questions (scored 0 to 8)* | |
| 1. Can you reach your case manager/care coordinator when you need to? | 0 = no, or only sometimes  1 = most of the time, usually  2 = yes, always |
| 2. If you want to make changes to your services, do you know whom to contact? | 0 = no  1 = not sure, maybe  2 = yes |
| 3. Can you choose or change what kind of services you get? | 0 = no  1 = sometimes, or some services  2 = yes, all services |
| 4. Can you choose or change how often and when you get your services?” | 0 = no  1 = sometimes, or some services  2 = yes, all services |

**Supplementary Table 2**. Survey Month

| **NCI-AD Survey Month** | **Full Sample**  **(N = 1,413)** | **Community-Dwelling**  **(N = 839)** | **Assisted Living**  **(N = 574)** |
| --- | --- | --- | --- |
| February 2018 | 270 (19%) | 145 (17%) | 125 (22%) |
| March 2018 | 457 (32%) | 264 (31%) | 193 (34%) |
| April 2018 | 375 (27%) | 227 (27%) | 148 (26%) |
| May 2018 | 264 (19%) | 167 (20%) | 97 (17%) |
| June 2018 | 47 (3.3%) | 36 (4.3%) | 11 (1.9%) |

**Supplementary Table 3.** Characteristics of Minnesota NCI-AD Respondents by Dementia Diagnosis

| **Characteristics** | **Full Sample**  **(*N* = 1,413)** | **No Dementia**  **(*n* = 931)** | **Dementia**  **(*n* = 482)** |
| --- | --- | --- | --- |
| Assisted living, n (%) | 574 (41%) | 282 (30%) | 292 (61%) |
| Age Group, n (%) |  |  |  |
| 65-74 | 411 (29%) | 309 (33%) | 102 (21%) |
| 75-84 | 548 (39%) | 361 (39%) | 187 (39%) |
| 85+ | 454 (32%) | 261 (28%) | 193 (40%) |
| Female, n (%) | 1,050 (74%) | 708 (76%) | 342 (71%) |
| Race, n (%) |  |  |  |
| African American / Black | 160 (11%) | 117 (13%) | 43 (8.9%) |
| White | 1,059 (75%) | 685 (74%) | 374 (78%) |
| Hispanic | 135 (9.6%) | 90 (9.7%) | 45 (9.3%) |
| Other | 59 (4.2%) | 39 (4.2%) | 20 (4.1%) |
| Marital Status, n (%) |  |  |  |
| Married | 130 (9.2%) | 89 (9.6%) | 41 (8.5%) |
| Unmarried | 1,221 (86%) | 810 (87%) | 411 (85%) |
| Unknown / refused | 62 (4.4%) | 32 (3.4%) | 30 (6.2%) |
| HCBS Service Utilization, Mean (SD%) |  |  |  |
| Home health | 0.29 (0.40) | 0.31 (0.41) | 0.24 (0.37) |
| DME/ modifications | 0.65 (0.38) | 0.66 (0.38) | 0.63 (0.37) |
| Non-medical Transport | 0.29 (0.37) | 0.31 (0.38) | 0.25 (0.34) |
| Personal care assistant | 0.17 (0.36) | 0.18 (0.37) | 0.14 (0.34) |
| Case management | 0.39 (0.36) | 0.39 (0.37) | 0.39 (0.35) |
| Home services | 0.43 (0.45) | 0.51 (0.46) | 0.27 (0.41) |
| Adult day | 0.10 (0.28) | 0.10 (0.28) | 0.09 (0.27) |
| Comorbidities, n (%) |  |  |  |
| Brain injury | 64 (4.5%) | 33 (3.5%) | 31 (6.4%) |
| Cancer | 236 (17%) | 155 (17%) | 81 (17%) |
| Congestive heart failure | 483 (34%) | 311 (33%) | 172 (36%) |
| COPD | 700 (50%) | 473 (51%) | 227 (47%) |
| Stroke | 443 (31%) | 252 (27%) | 191 (40%) |
| Depression | 762 (54%) | 471 (51%) | 291 (60%) |
| Diabetes | 678 (48%) | 450 (48%) | 228 (47%) |
| Hard of hearing | 564 (40%) | 366 (39%) | 198 (41%) |
| Hypertension | 1,240 (88%) | 812 (87%) | 428 (89%) |
| Liver disease | 180 (13%) | 117 (13%) | 63 (13%) |
| Mental health | 990 (70%) | 616 (66%) | 374 (78%) |
| Myocardial infarction | 263 (19%) | 177 (19%) | 86 (18%) |
| Obesity | 534 (38%) | 377 (40%) | 157 (33%) |
| Peripheral vascular disease | 613 (43%) | 387 (42%) | 226 (47%) |
| Kidney disease | 484 (34%) | 301 (32%) | 183 (38%) |
| Serious mental illness | 158 (11%) | 97 (10%) | 61 (13%) |
| Quality, mean (SD) |  |  |  |
| Overall | 0.85 (0.14) | 0.86 (0.14) | 0.83 (0.16) |
| Security domain | 0.89 (0.19) | 0.89 (0.18) | 0.87 (0.22) |
| Self-determination domain | 0.85 (0.17) | 0.85 (0.17) | 0.84 (0.18) |
| Care experience domain | 0.79 (0.27) | 0.81 (0.26) | 0.76 (0.29) |

**Supplementary Table 4.** Mean (SD) Overall Quality for People without and with a Proxy Respondent

| **Dementia Status** | **Overall**  **(*N* = 1,413)** | **Self**  **(*n* = 1,332)** | **Proxy**  **(*n* = 81)** |
| --- | --- | --- | --- |
| No Dementia | 0.86 (0.14) | 0.86 (0.13) | 0.82 (0.23) |
| Dementia | 0.83 (0.16) | 0.82 (0.16) | 0.90 (0.15) |

**Supplementary Table 5**. Adjusted average differences in quality score for service use in all months vs no service use (averaged across dementia status and covariates).

| **Variable** | **Community Dwelling**  **(N=839)** | **Assisted Living Residents**  **(N=574)** |
| --- | --- | --- |
|  | **Adjusted Mean Difference** | **Adjusted Mean Difference** |
| Home Health Services | -0.01 (-0.04, 0.01); p=0.34 | 0.04 (-0.03, 0.11); p=0.21 |
| Durable Medical Equipment/Home Modifications^1^ | -0.01 (-0.03, 0.01); p=0.37 | 0.03 (0.00, 0.07); p=0.033* |
| Non-medical Transportation | -0.01 (-0.03, 0.02); p=0.56 | -0.04 (-0.09, 0.01); p=0.14 |
| Personal Care Assistant Services | 0.02 (0.00, 0.05); p=0.022* | NA |
| Case Management | 0.02 (-0.02, 0.07); p=0.38 | -0.02 (-0.09, 0.04); p=0.46 |
| Homemaker Services | 0.01 (-0.02, 0.04); p=0.55 | -0.03 (-0.09, 0.03); p=0.34 |
| Adult day care | 0.02 (-0.02, 0.06); p=0.27 | NA |
| *^1^**p<0.05; **p<0.01; ***p<0.001 | | |

Note. ^1^People in assisted living received durable medical equipment but not home modifications

**Supplementary Figure 1.** Community-Dwelling Respondents: Adjusted Means and Mean Differences on the Care Experience Domain


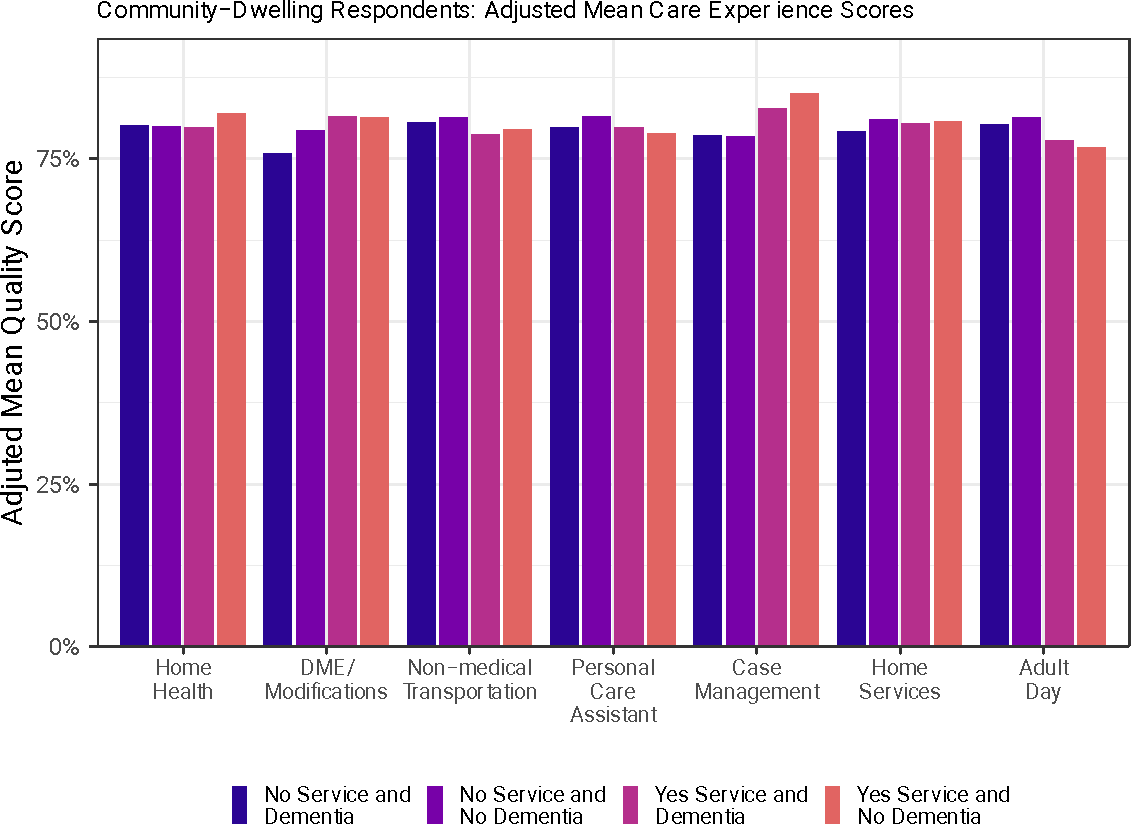

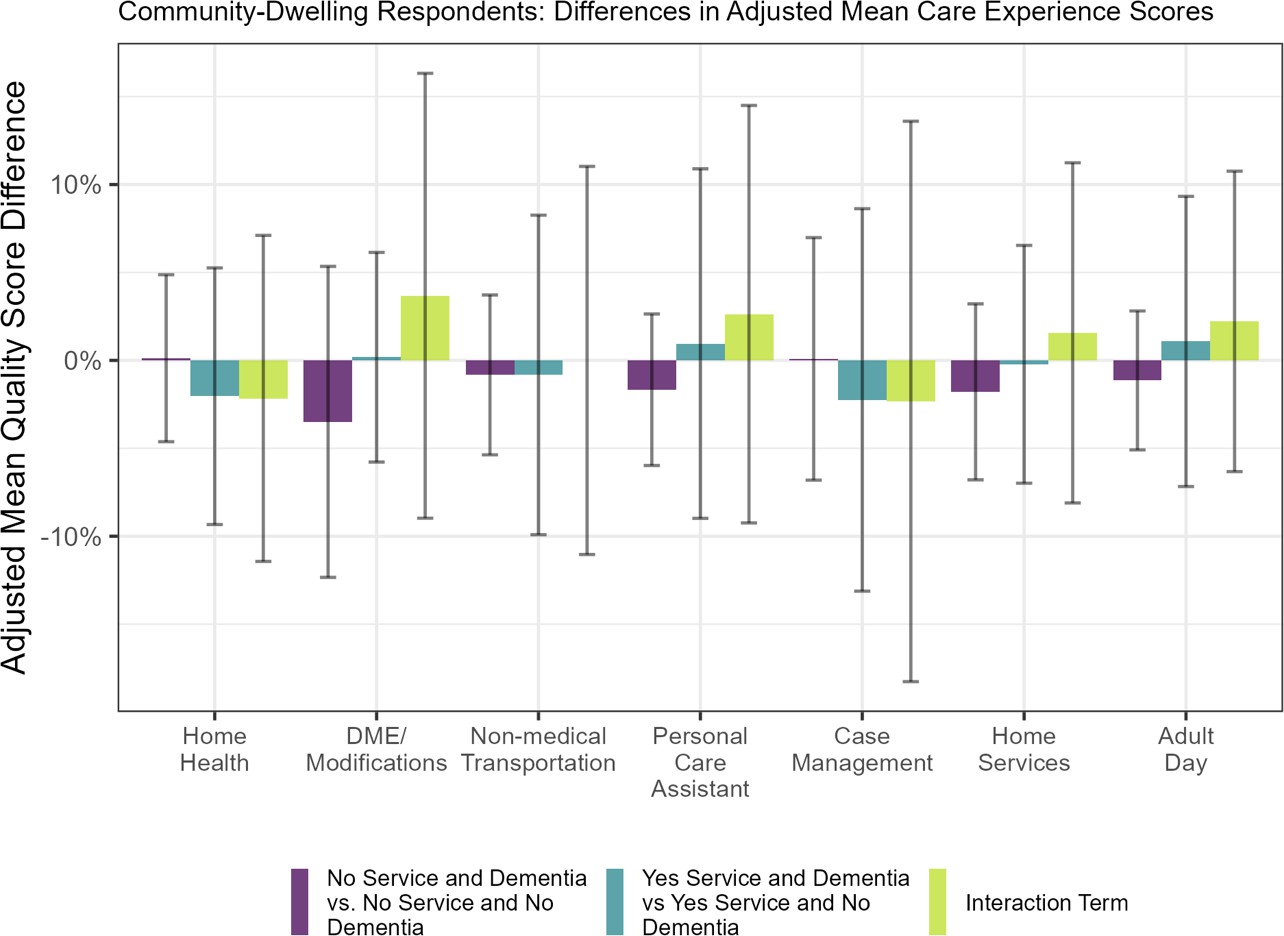


**Supplementary Figure 2.** Community-Dwelling Respondents: Adjusted Means and Mean Differences on the Security Domain


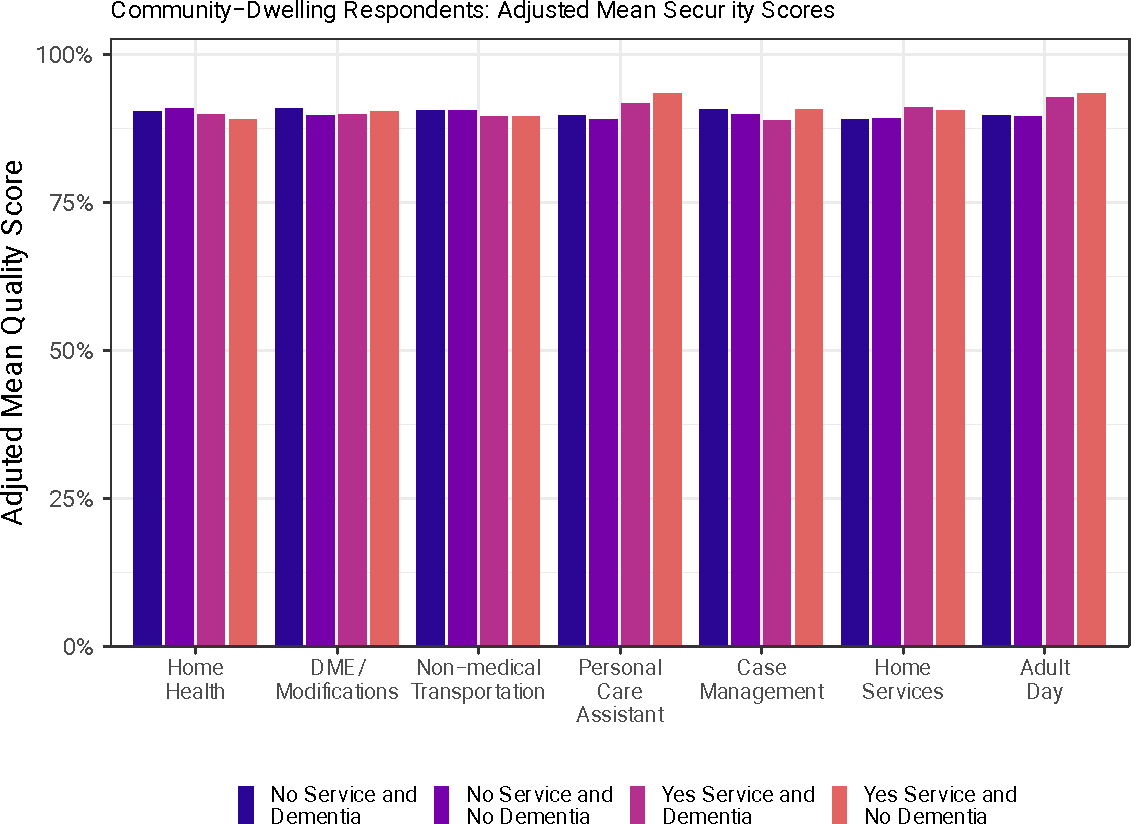

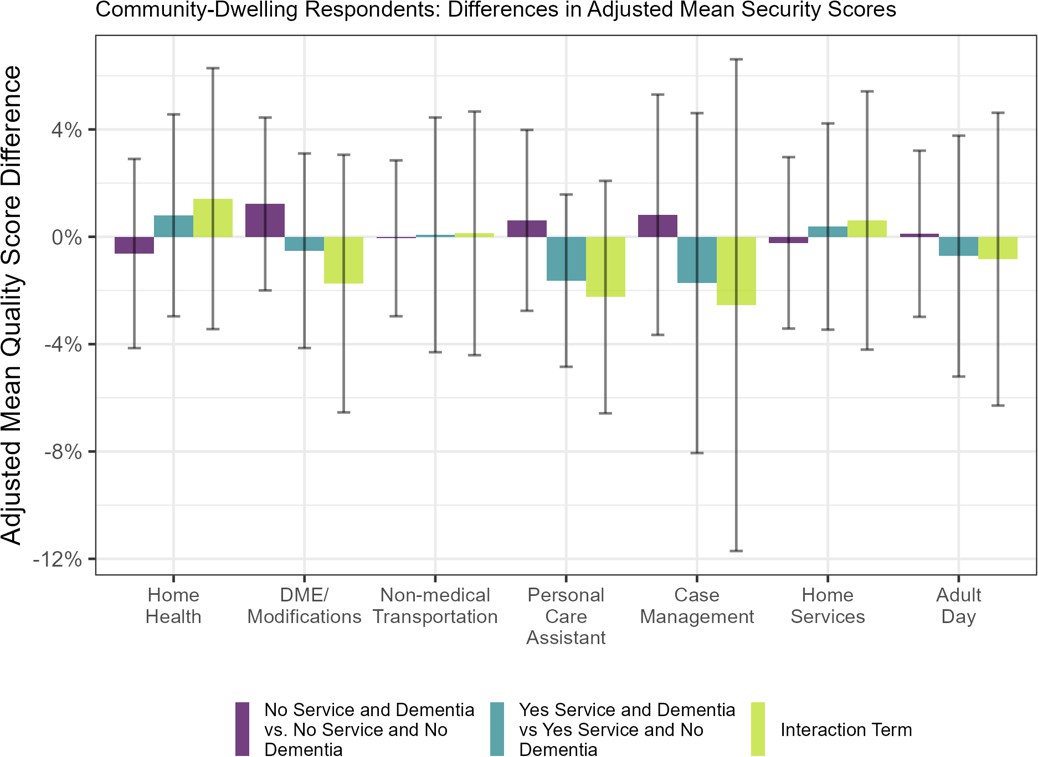


**Supplementary Figure 3.** Community-Dwelling Respondents: Adjusted Means and Mean Differences on Self-determination Domain


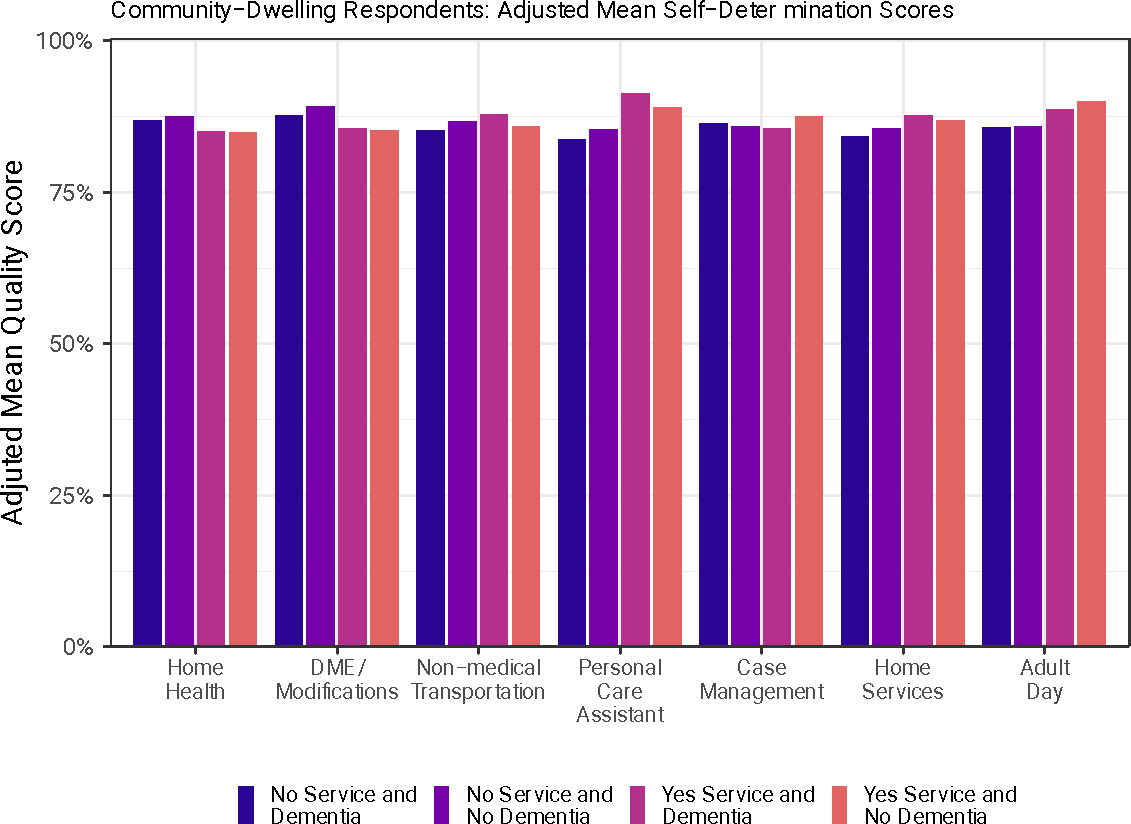

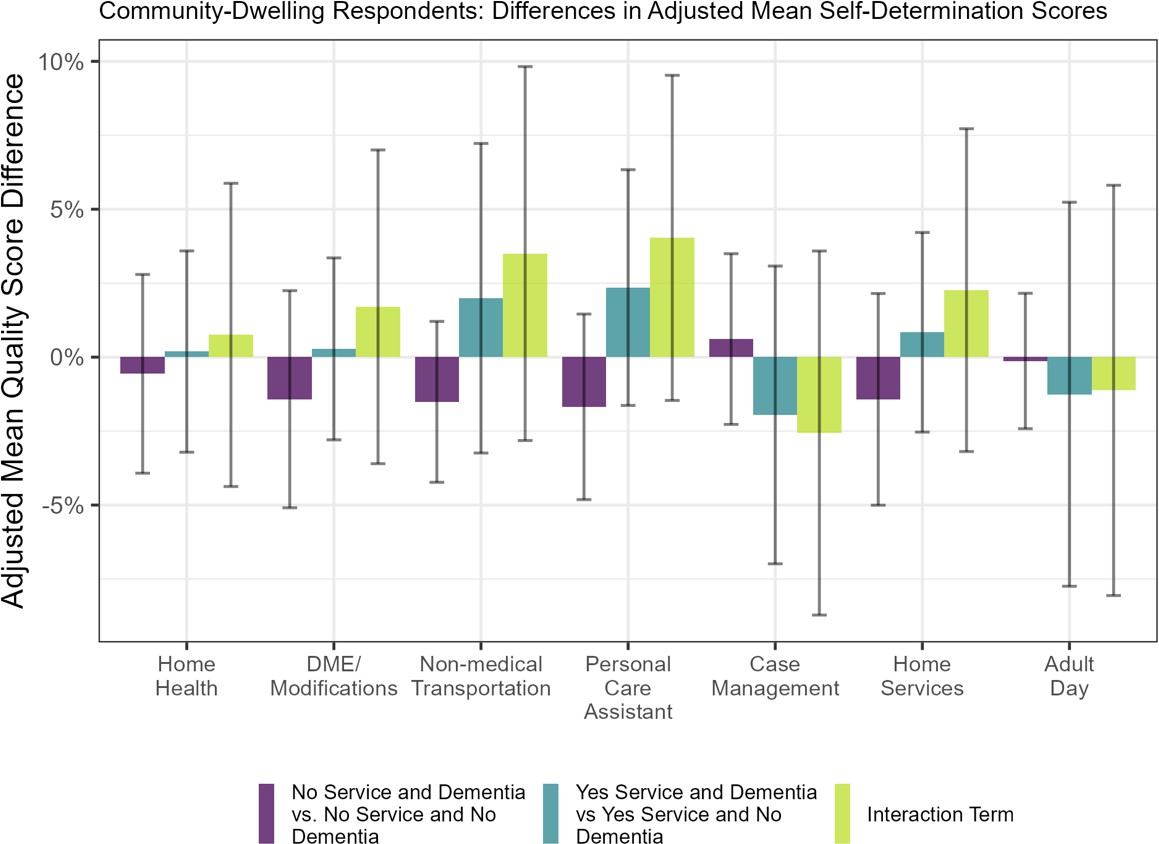


**Supplementary Figure 4.** Assisted Living Respondents: Adjusted Means and Mean Differences on the Care Experience Domain


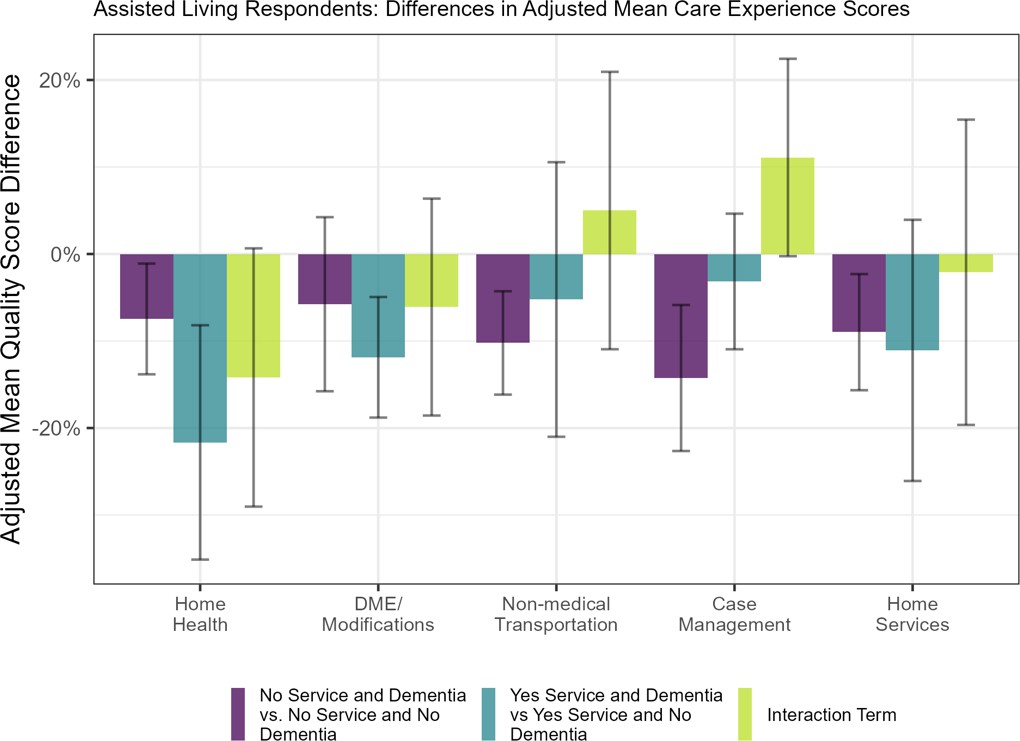

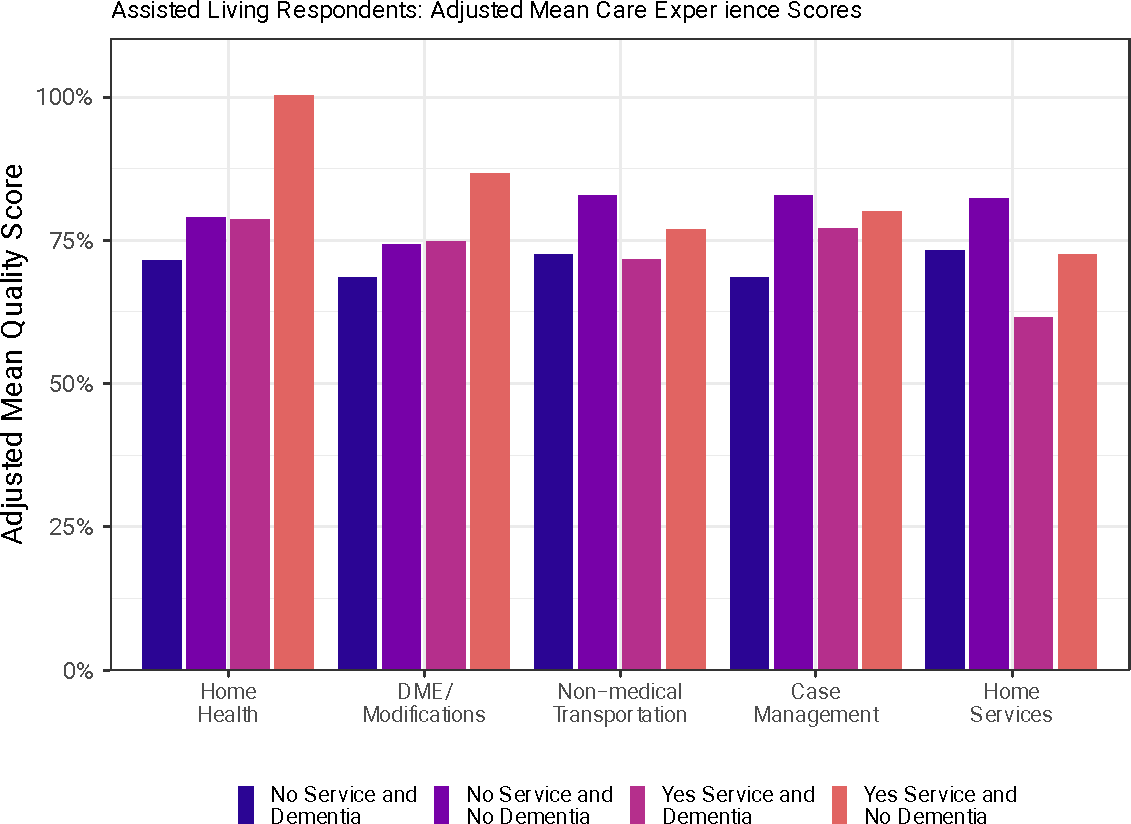


**Supplementary Figure 5.** Assisted Living Respondents: Adjusted Means and Mean Differences on the Security Domain


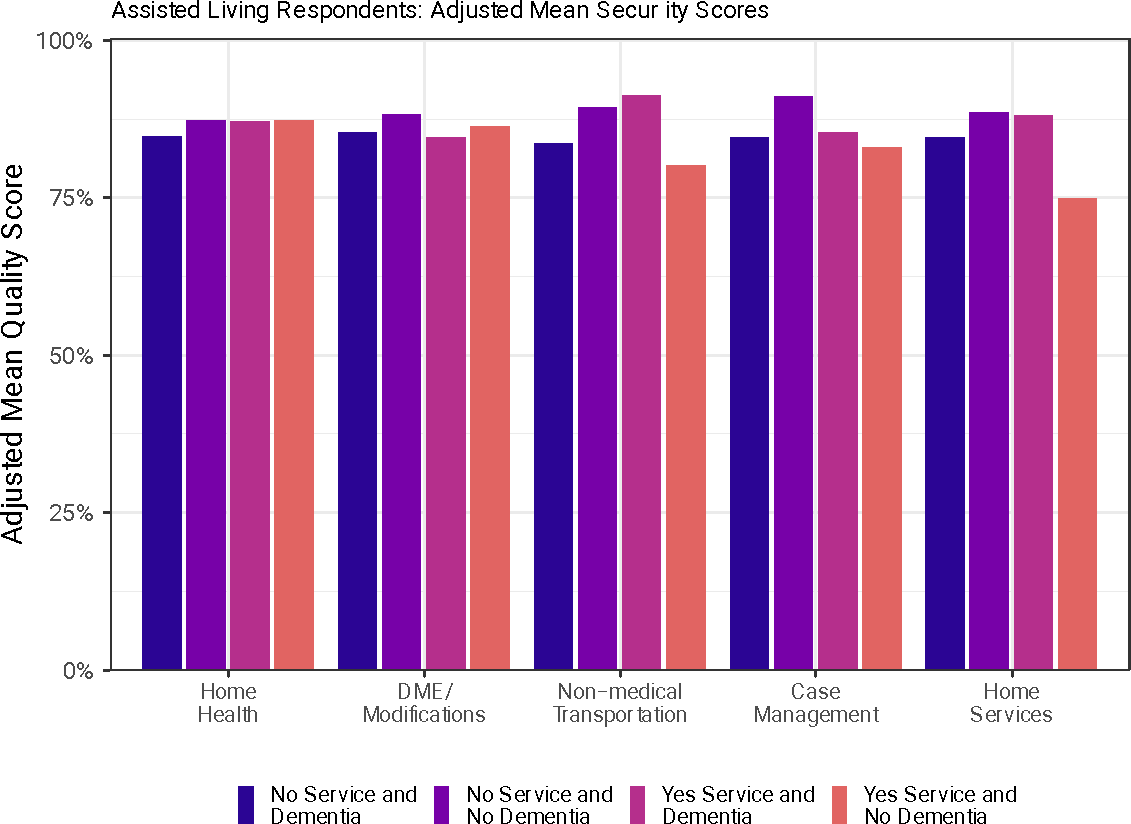

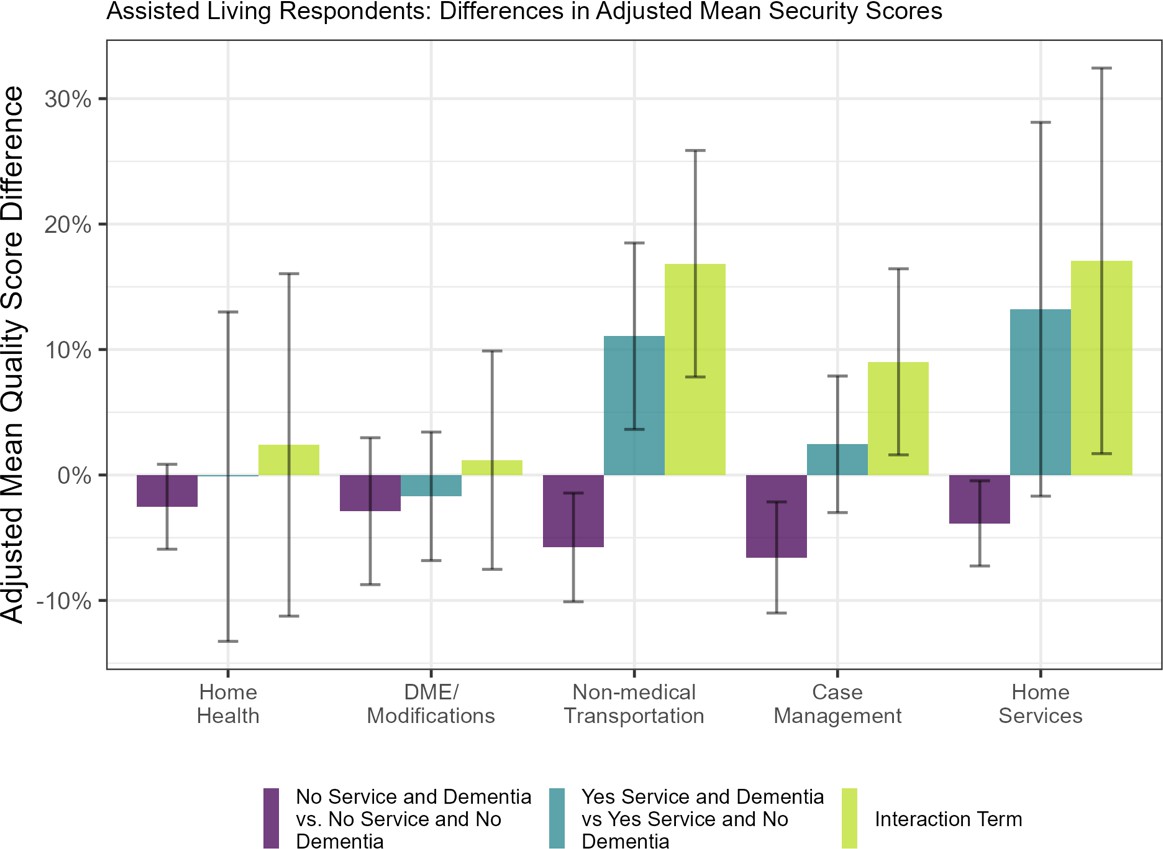


**Supplementary Figure 6.** Assisted Living Respondents: Adjusted Means and Mean Differences on the Self-determination Domain


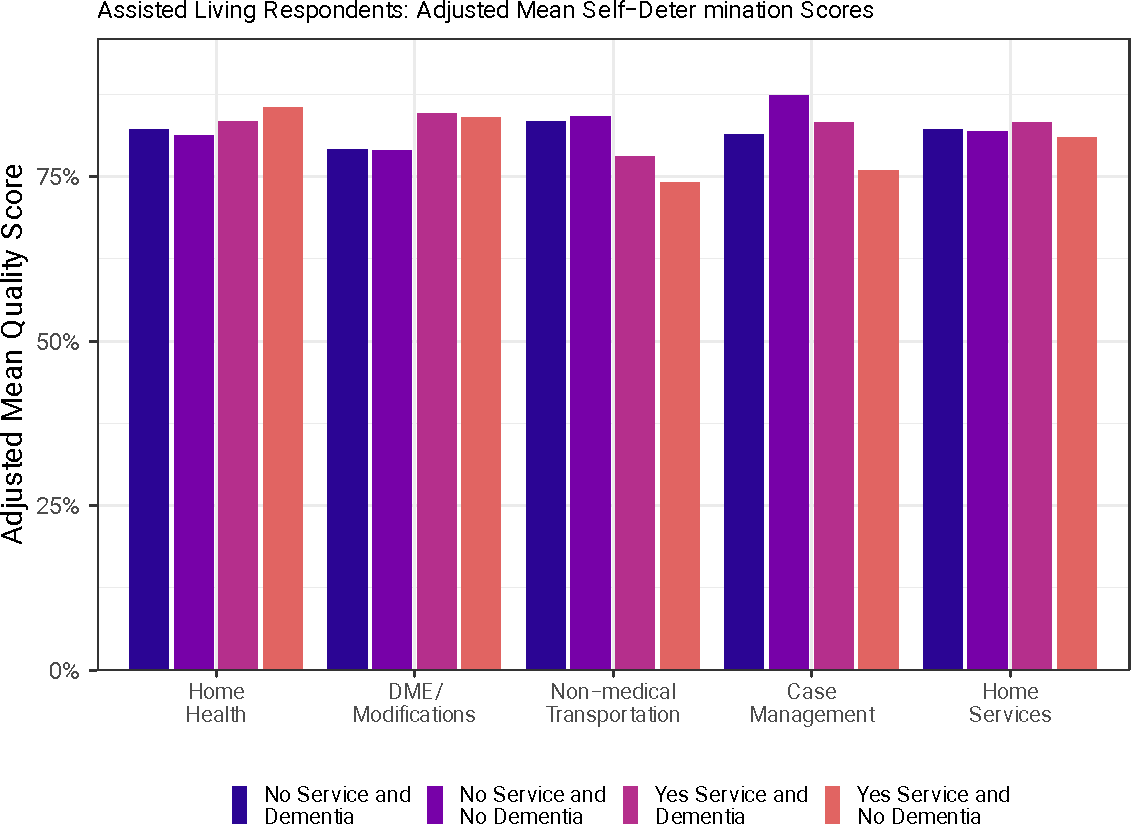

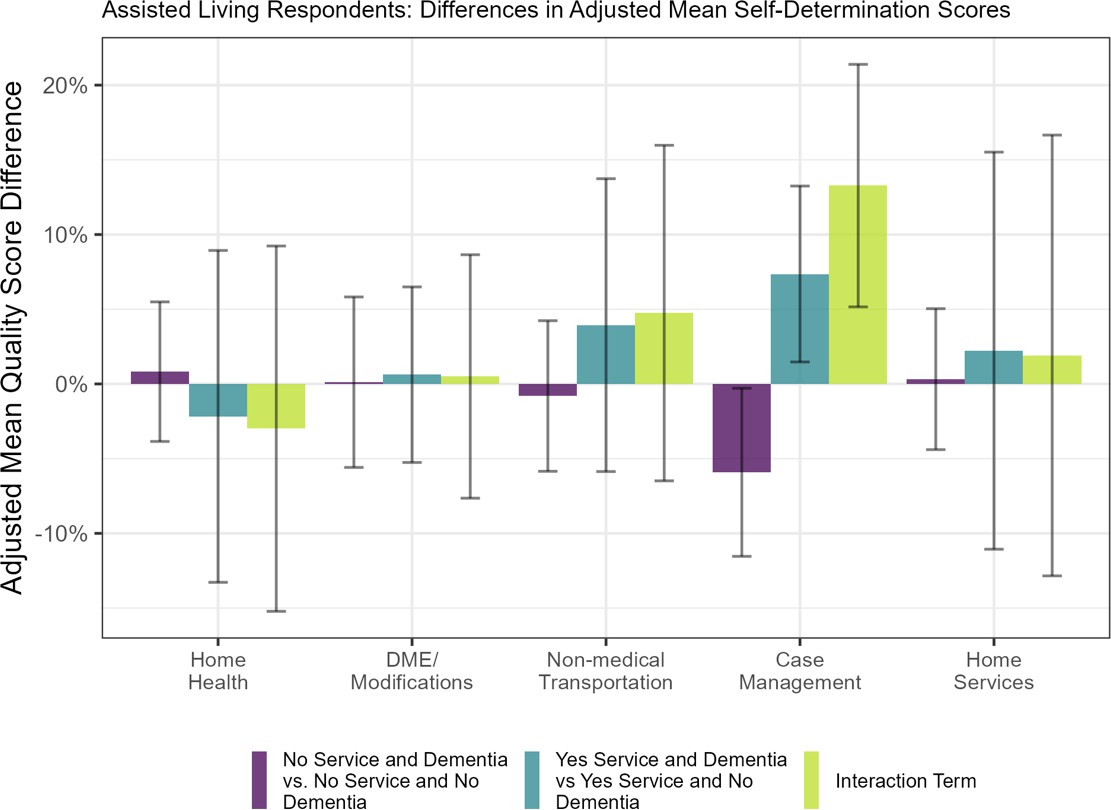

Supplement: igaf118_Supplementary_Data [file igaf118_supplementary_data.docx]
